# Short- and Long-Term Outcomes of Robot-Assisted Versus Video-Assisted Thoracoscopic Esophagectomy for Esophageal and Esophagogastric Junction Cancer

**DOI:** 10.3390/cancers18142246

**Published:** 2026-07-14

**Authors:** Eisuke Booka, Takahiro Kuroda, Shuhei Yasumoto, Keita Misu, Yuki Sakai, Kenichi Sekimori, Shunta Nakamura, Ryoma Haneda, Wataru Soneda, Mayu Sakata, Yoshifumi Morita, Hirotoshi Kikuchi, Yoshihiro Hiramatsu, Hiroya Takeuchi

**Affiliations:** 1 Department of Surgery, Hamamatsu University School of Medicine, Hamamatsu 431-3192, Shizuoka, Japan; booka@hama-med.ac.jp (E.B.); 41245561@hama-med.ac.jp (S.Y.); hiramatu@hama-med.ac.jp (Y.H.); 2 Division of Surgical Care, Morimachi, Hamamatsu University School of Medicine, Hamamatsu 431-3192, Shizuoka, Japan; 3 Department of Perioperative Functioning Care and Support, Hamamatsu University School of Medicine, Hamamatsu 431-3192, Shizuoka, Japan

**Keywords:** esophageal cancer, esophagogastric junction cancer, robot-assisted minimally invasive esophagectomy, video-assisted thoracoscopic esophagectomy, recurrent laryngeal nerve paralysis, propensity score matching, overall survival, recurrence-free survival

## Abstract

Background/Objectives: Thoracoscopic esophagectomy is an accepted minimally invasive approach for resectable esophageal and esophagogastric junction cancer in Japan. Whether robot-assisted minimally invasive esophagectomy (RAMIE) improves short- and long-term outcomes compared with video-assisted thoracoscopic esophagectomy (VATS) remains clinically relevant. Methods: We retrospectively reviewed patients who underwent VATS or RAMIE between January 2017 and April 2026. Patients who underwent open esophagectomy, non-gastric conduit reconstruction, or staged operation were excluded. Propensity score matching (PSM) was performed using age, sex, body mass index, histology, tumor location, clinical stage, preoperative therapy, extent of lymph node dissection, abdominal approach, and reconstruction route. Long-term outcomes were explored in a PSM cohort limited to patients treated between January 2017 and December 2023. Results: The main cohort included 369 patients (VATS, *n* = 186; RAMIE, *n* = 183). Overall complications of Clavien–Dindo (CD) grade ≥ II were comparable between VATS and RAMIE (32.8% vs. 32.8%, *p* = 0.999), whereas RLNP CD ≥ I (9.7% vs. 3.8%, *p* = 0.025) and RLNP CD ≥ II (3.8% vs. 0.5%, *p* = 0.034) were less frequent after RAMIE. PSM yielded 128 matched pairs; RLNP remained less frequent after RAMIE (CD ≥ I, 10.9% vs. 3.1%, *p* = 0.015; CD ≥ II, 4.7% vs. 0%, *p* = 0.013), while other short-term outcomes were comparable. In the survival PSM cohort (79 pairs), 3-year overall survival (66.0% vs. 73.4%, *p* = 0.203) and recurrence-free survival (62.3% vs. 59.3%, *p* = 0.651) did not differ significantly between VATS and RAMIE. Conclusions: RAMIE showed comparable short-term safety to VATS and was associated with lower RLNP after adjustment for clinicopathological, treatment-related, and surgical factors. Exploratory long-term analysis was limited by cohort size and follow-up duration but suggested that RAMIE did not compromise oncologic outcomes.

## Simple Summary

Esophagectomy for esophageal and esophagogastric junction cancer is technically demanding and may cause recurrent laryngeal nerve paralysis (RLNP). In Japan, thoracoscopic esophagectomy has become a standard minimally invasive approach, and the next clinical question is whether robotic assistance adds further benefit. We compared robot-assisted minimally invasive esophagectomy (RAMIE) with video-assisted thoracoscopic esophagectomy (VATS) in patients who underwent one-stage gastric conduit reconstruction. RAMIE showed comparable short-term safety and a lower incidence of RLNP. After propensity score matching using clinicopathological and treatment-related factors, including preoperative therapy and two- and three-field lymph node dissection, RLNP remained less frequent after RAMIE. In an exploratory matched survival cohort, overall and recurrence-free survival did not differ significantly between groups. These findings suggest that RAMIE is a safe alternative to VATS and may reduce RLNP without compromising oncologic outcomes, even though early RAMIE implementation cases were included; the limited survival cohort still requires cautious interpretation.

## 1. Introduction

Esophageal cancer remains a major cause of cancer-related mortality worldwide, and esophagectomy with radical lymphadenectomy remains a key component of curative treatment for localized disease [1]. Esophageal squamous cell carcinoma predominates in East Asia, whereas adenocarcinoma and esophagogastric junction cancer are increasingly encountered in Western countries and in Japan. Curative treatment therefore requires careful integration of tumor location, histology, clinical stage, preoperative therapy, and surgical quality. However, transthoracic esophagectomy is one of the most invasive gastrointestinal procedures, and postoperative complications can delay recovery, impair quality of life, and adversely affect long-term outcomes [2].

Minimally invasive esophagectomy (MIE) has been introduced to reduce surgical trauma while maintaining oncologic quality [3,4,5]. The Japanese JCOG1409 MONET trial, a multicenter phase 3 trial comparing thoracoscopic and open esophagectomy with overall survival as the primary endpoint, confirmed the non-inferiority of thoracoscopic esophagectomy and supported its role as a standard treatment in Japan [3]. Earlier randomized trials also showed short-term benefits of minimally invasive or hybrid minimally invasive approaches compared with open surgery [4,5]. Consequently, in settings where video-assisted thoracoscopic esophagectomy (VATS) is established, the relevant clinical question has shifted from whether minimally invasive surgery is acceptable to whether robot assistance can improve selected outcomes over conventional thoracoscopy.

Robot-assisted minimally invasive esophagectomy (RAMIE) offers magnified three-dimensional vision, articulated instruments, stable camera control, and tremor filtration [6]. These features may be particularly useful during upper mediastinal lymphadenectomy along the recurrent laryngeal nerves, where traction, compression, and thermal injury may cause recurrent laryngeal nerve paralysis (RLNP). Recent randomized evidence has begun to address RAMIE versus conventional MIE or VATS. The RAMIE trial reported comparable perioperative safety with shorter operative duration and improved feasibility of recurrent laryngeal nerve lymphadenectomy in selected subgroups [7]. The REVATE trial directly compared robot-assisted and video-assisted thoracoscopic esophagectomy and showed improved dissection success along the left recurrent laryngeal nerve (RLN) and less postoperative RLNP after robotic surgery [8]. Long-term follow-up from the RAMIE trial also suggested that RAMIE is at least non-inferior to thoracoscopic MIE in survival [9].

Although these randomized trials provide high-level evidence, additional real-world data remain useful in settings where VATS has already been established as a standard operation and where detailed institutional standardization of RAMIE has occurred. Our previous single-institution study compared open esophagectomy, VATS, and RAMIE and suggested that RAMIE could reduce postoperative complications; however, the RAMIE cohort was limited to 35 patients, and the study mixed open and minimally invasive procedures [10]. We hypothesized that RAMIE would reduce the incidence of RLNP compared with VATS without compromising long-term oncologic outcomes. Therefore, the present study was designed as an updated analysis focused specifically on patients undergoing VATS or RAMIE with one-stage gastric conduit reconstruction, with special attention to RLNP, overall short-term safety, and exploratory long-term oncologic outcomes.

## 2. Materials and Methods

### 2.1. Study Design and Patients

This retrospective single-institution study reviewed consecutive patients who underwent esophagectomy for esophageal or esophagogastric junction cancer at Hamamatsu University School of Medicine between January 2017 and April 2026. In this study, VATS and RAMIE refer to the thoracic approach. VATS had been established at our institution before the study period, whereas RAMIE was introduced during the study period after structured preparation, team training, and procedural standardization. The choice between VATS and RAMIE was based on patient- and tumor-related factors, institutional surgical scheduling, robotic system availability, and surgeon judgment; it was not randomized. All procedures were performed or supervised by experienced esophageal surgeons in a limited institutional surgical team. The abdominal phase was performed either by open surgery or laparoscopy, and the abdominal approach was included as a covariate in the propensity score model.

**Figure 1:**
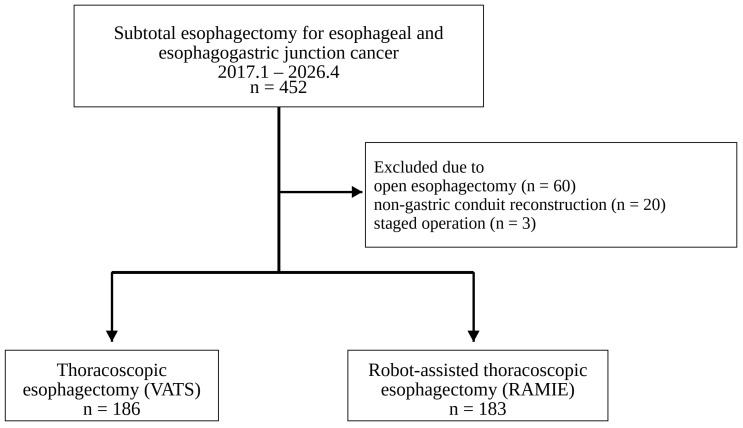
Study flow diagram.

Among 452 patients who underwent subtotal esophagectomy for esophageal or esophagogastric junction cancer during the study period, 60 patients who underwent open esophagectomy were excluded. The remaining source dataset contained 392 VATS/RAMIE cases (VATS, *n* = 197; RAMIE, *n* = 195). We further excluded 20 patients who underwent non-gastric conduit reconstruction and three patients who underwent staged operations. The final main cohort therefore comprised 369 patients: 186 in the VATS group and 183 in the RAMIE group. This restriction was intended to reduce heterogeneity related to reconstruction, because the reconstruction route and anastomotic procedure can influence short-term outcomes after esophagectomy. The study flow is shown in Figure 1.

Clinical stage was classified according to the 8th edition of the TNM classification [11]. The extent of lymph node dissection was categorized as two-field lymph node dissection (2FLND) or three-field lymph node dissection (3FLND) according to Japanese guidelines and the Japanese Classification of Esophageal Cancer [12,13,14,15]. Preoperative therapy was categorized as upfront surgery, preoperative chemotherapy, or preoperative chemoradiotherapy. For statistical categorization, patients who received systemic chemotherapy before surgery for initially unresectable or borderline resectable disease were included in the preoperative chemotherapy category, and patients who underwent surgery after definitive chemoradiotherapy were included in the preoperative chemoradiotherapy category. These categories were used for baseline adjustment and do not imply equivalence between standard preoperative treatment and surgery performed after induction chemotherapy or definitive chemoradiotherapy. When surgery was judged feasible, mediastinal lymphadenectomy was performed according to the same institutional surgical principles, but technical difficulty after definitive chemoradiotherapy could not be fully captured in this retrospective dataset.

### 2.2. Surgical Procedures

All patients underwent transthoracic esophagectomy with gastric conduit reconstruction as a one-stage procedure. Lymphadenectomy along the bilateral recurrent laryngeal nerves was performed in all patients. In general, 3FLND included cervical, mediastinal, and abdominal lymphadenectomy, whereas 2FLND included mediastinal and abdominal lymphadenectomy; cervical lymphadenectomy was performed according to tumor location and institutional policy. VATS was performed using a thoracoscopic approach, and RAMIE was performed using either the da Vinci Xi or hinotori robotic system. The standardized institutional RAMIE procedure in the semi-prone position, including patient positioning, port placement, mediastinal lymphadenectomy, and reconstruction strategy, has been described previously [16]. In brief, upper mediastinal lymphadenectomy was performed with careful exposure of the bilateral RLNs, preservation of the nerve in its original position as much as possible, early division of esophageal branches when appropriate, and minimization of traction, compression, and thermal spread. During VATS, mediastinal dissection was mainly performed using a LigaSure vessel-sealing system, electrocautery, clips, scissors, and suction retractors according to the operative field. During RAMIE, Maryland bipolar forceps, vessel-sealing devices, Potts scissors, clips, and suction retractors were used according to the operative field. In both approaches, energy-device use around the RLN was minimized when necessary, and the basic nerve-preserving dissection concept was shared between VATS and RAMIE. Intraoperative nerve monitoring was not used in any patient. Robotic surgery provided articulated instrumentation and stable three-dimensional visualization. The same standardized mediastinal dissection concept was applied across robotic platforms; platform-specific subgroup analysis was not performed because of the limited number of cases on the newer platform. The same institutional postoperative pathway, including intensive-care and ward management, analgesia, respiratory care, nutritional support, rehabilitation, laryngoscopic assessment, oral-intake progression, and complication management, was used for VATS and RAMIE regardless of abdominal approach or robotic platform.

### 2.3. Outcomes

The primary endpoint was RLNP CD ≥ I. Secondary endpoints included RLNP CD ≥ II, overall postoperative complications CD ≥ II, severe postoperative complications CD ≥ III, anastomotic leakage CD ≥ II, pneumonia CD ≥ II, surgical site infection CD ≥ II, operative time, blood loss, and postoperative hospital stay. Exploratory long-term endpoints were overall survival (OS) and recurrence-free survival (RFS). Postoperative complications were graded according to the Clavien–Dindo classification [17]. Postoperative vocal cord function was routinely assessed by laryngoscopy on postoperative day 7 in all patients, and the examination was performed by rehabilitation physicians according to institutional practice. Impaired vocal cord movement on laryngoscopy was defined as Grade I RLNP. Grade II RLNP was defined as aspiration requiring medical treatment such as antibiotics. Grade IIIa RLNP was defined as aspiration severe enough to preclude oral intake and requiring treatment under local anesthesia, and Grade IIIb RLNP was defined as aspiration requiring treatment under general anesthesia. For RLNP, Grade I or higher was defined as CD ≥ I, and Grade II or higher was defined as CD ≥ II.

### 2.4. Statistical Analysis

Continuous variables are presented as medians with interquartile ranges and were compared using the Mann–Whitney U test. Categorical variables are presented as numbers and percentages and were compared using Pearson’s chi-square test or Fisher’s exact test, as appropriate. A sensitivity analysis was performed according to the extent of lymph node dissection. PSM was performed to address imbalance between the VATS and RAMIE groups. The propensity score model included age, sex, body mass index (BMI), histology, tumor location, clinical stage (0–II vs. III–IV according to TNM 8th), preoperative therapy, extent of lymph node dissection, abdominal approach, and reconstruction route. Matching was performed using 1:1 nearest-neighbor matching without replacement with a caliper of 0.2 standard deviations of the logit of the propensity score. Balance was assessed using standardized mean differences (SMDs). After matching, continuous variables were compared using the Mann–Whitney U test and categorical variables using Pearson’s chi-square test or Fisher’s exact test, as appropriate. For exploratory long-term analysis, a survival cohort was defined by limiting patients to those treated between January 2017 and December 2023, and PSM was repeated using the same covariates. OS was calculated from the date of surgery to death from any cause, and RFS was calculated from the date of surgery to recurrence or death. Survival curves were estimated using the Kaplan–Meier method and compared using the log-rank test. The survival analysis was exploratory and not powered to demonstrate oncologic equivalence. Two-sided *p* values <0.05 were considered statistically significant. Additional exploratory cStage-stratified survival analyses were performed in the overall survival cohort by grouping patients into cStage 0/I/II and cStage III/IV; these analyses were not separately propensity score-matched within each stage stratum and were considered hypothesis-generating.

OpenAI ChatGPT (GPT-5.5 Pro, accessed in June 2026) was used as an AI-assisted tool to help generate preliminary statistical summaries, organize propensity score-matching tables, and prepare Kaplan–Meier figures from the author-provided dataset. All data coding, clinical definitions, statistical decisions, numerical results, and interpretations were verified and finalized by the authors.

## 3. Results

### 3.1. Patient Selection and Baseline Characteristics

Baseline characteristics before and after propensity score matching are summarized in Table 1. A total of 369 patients met the inclusion criteria for the main analysis (VATS, *n* = 186; RAMIE, *n* = 183). The median age was similar between the VATS and RAMIE groups (69 vs. 70 years, *p* = 0.569), and sex distribution was also comparable. Median BMI tended to be slightly higher in the RAMIE group (21.8 vs. 21.2 kg/m^2^, *p* = 0.059). Preoperative therapy was balanced between groups (*p* = 0.949).

The RAMIE group contained more clinically advanced cases and fewer 3FLND procedures. Clinical stage III–IV disease was observed in 52.5% of the RAMIE group and 43.0% of the VATS group (*p* = 0.069), and 3FLND was performed less frequently in RAMIE than in VATS (25.1% vs. 46.2%, *p* < 0.001).

### 3.2. Short-Term Surgical Outcomes

Intraoperative and postoperative outcomes are shown in Table 2. Overall postoperative morbidity was comparable between the groups. Overall complications CD ≥ II occurred in 32.8% of both the VATS and RAMIE groups (*p* = 0.999). Severe complications CD ≥ III occurred in 8.6% and 12.0%, respectively (*p* = 0.280). There were no significant differences in anastomotic leakage CD ≥ II, pneumonia CD ≥ II, surgical site infection CD ≥ II, operative time, blood loss, or postoperative hospital stay.

RLNP CD ≥ I was less frequent after RAMIE than after VATS (VATS vs. RAMIE, 9.7% vs. 3.8%; *p* = 0.025). RLNP CD ≥ II was also less frequent after RAMIE (3.8% vs. 0.5%; *p* = 0.034).

### 3.3. Propensity Score-Matched and Stratified Analyses for RLNP

PSM was performed to further assess the relationship between surgical approach and RLNP while balancing clinicopathological, treatment-related, and surgical factors, including preoperative therapy and the extent of LND. The PSM analysis yielded 128 matched pairs with adequate balance in baseline variables (Table 1). The propensity score-matched short-term outcomes are shown in Table 3. The distribution of 3FLND was balanced after matching (VATS, 44/128 [34.4%]; RAMIE, 40/128 [31.2%]; SMD = 0.067). In the matched cohort, RAMIE remained associated with lower incidences of RLNP CD ≥ I (VATS vs. RAMIE, 10.9% vs. 3.1%; *p* = 0.015) and RLNP CD ≥ II (4.7% vs. 0%; *p* = 0.013), whereas overall complications and other short-term outcomes were comparable.

In the PSM cohort, RLNP was further evaluated according to the extent of lymph node dissection (Table 4). Among patients undergoing 3FLND, RLNP CD ≥ I occurred in 10 of 44 VATS patients (22.7%) and one of 40 RAMIE patients (2.5%; *p* = 0.006), and RLNP CD ≥ II occurred in six of 44 VATS patients (13.6%) and 0 of 40 RAMIE patients (0%; *p* = 0.015). Among patients undergoing 2FLND, RLNP CD ≥ I was similar between VATS and RAMIE (4/84 [4.8%] vs. 3/88 [3.4%]; *p* = 0.654).

### 3.4. Exploratory Long-Term Outcomes in the Propensity Score-Matched Survival Cohort

**Figure 2:**
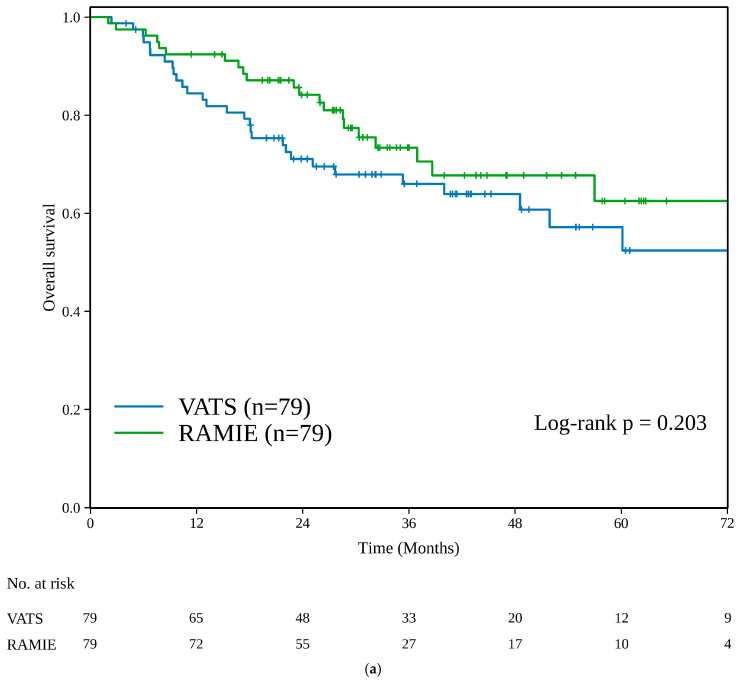

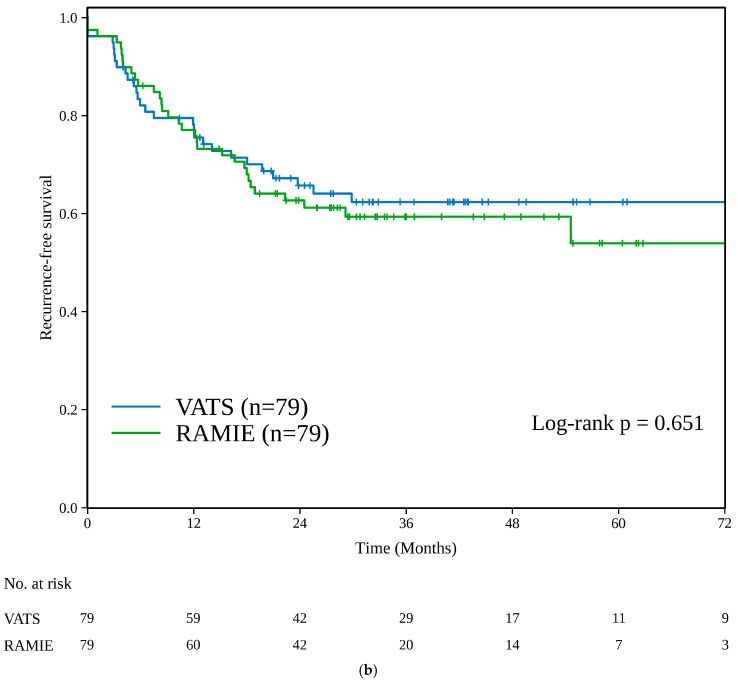
(**a**) Kaplan–Meier curve for overall survival in the propensity score-matched survival cohort. The survival cohort was limited to patients treated between January 2017 and December 2023 and included 79 matched pairs. (**b**) Kaplan–Meier curve for recurrence-free survival in the propensity score-matched survival cohort. The survival cohort was limited to patients treated between January 2017 and December 2023 and included 79 matched pairs.

For exploratory long-term outcome analysis, patients treated between January 2017 and December 2023 were selected, and PSM was repeated using the same covariates as in the short-term PSM analysis. This survival cohort comprised 79 matched pairs (VATS, *n* = 79; RAMIE, *n* = 79). OS events occurred in 29 VATS patients and 21 RAMIE patients. The 3-year OS rates were 66.0% in the VATS group and 73.4% in the RAMIE group, with no significant difference between groups (log-rank *p* = 0.203; Figure 2a). RFS events occurred in 28 VATS patients and 32 RAMIE patients. The 3-year RFS rates were 62.3% in the VATS group and 59.3% in the RAMIE group, with no significant difference between groups (log-rank *p* = 0.651; Figure 2b). Because RAMIE was introduced later than VATS and the survival cohort was limited in size, these survival curves should be interpreted as exploratory rather than confirmatory.

**Figure 3:**
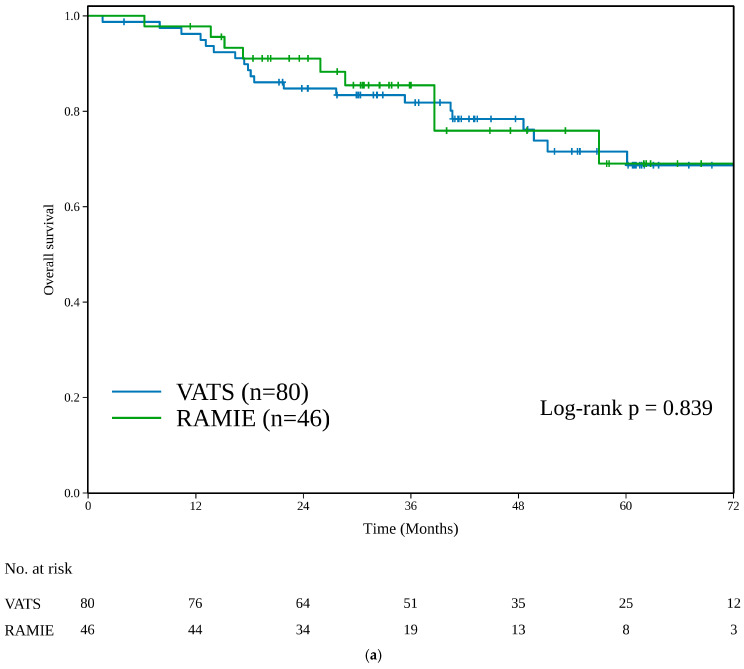

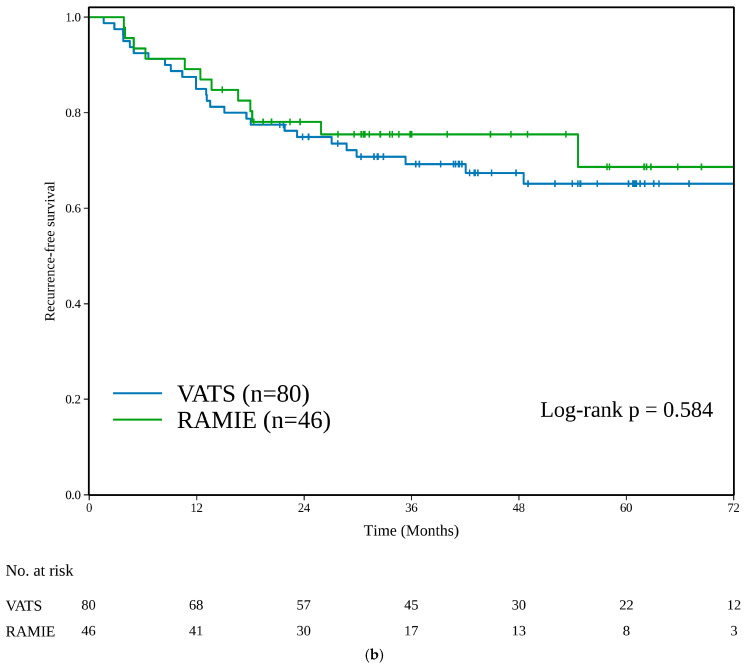
(**a**) Kaplan–Meier curve for overall survival in patients with cStage 0/I/II disease. The analysis was performed in the overall survival cohort limited to patients treated between January 2017 and December 2023. RAMIE, robot-assisted minimally invasive esophagectomy; VATS, video-assisted thoracoscopic esophagectomy. (**b**) Kaplan–Meier curve for recurrence-free survival in patients with cStage 0/I/II disease. The analysis was performed in the overall survival cohort limited to patients treated between January 2017 and December 2023. RAMIE, robot-assisted minimally invasive esophagectomy; VATS, video-assisted thoracoscopic esophagectomy.

**Figure 4:**
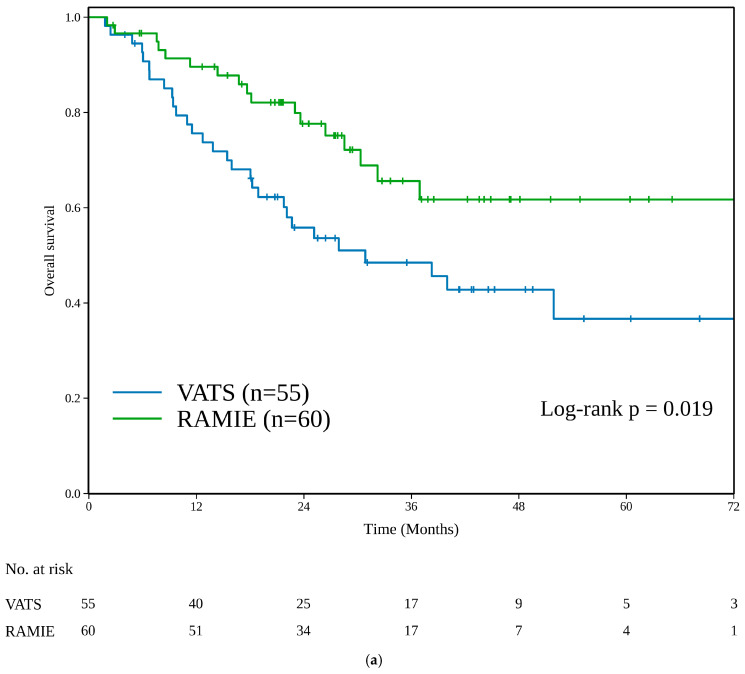

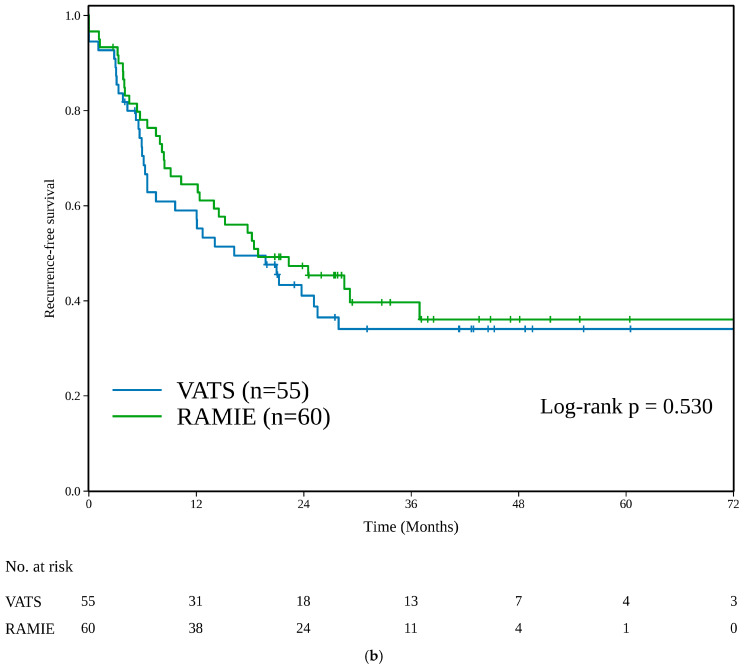
(**a**) Kaplan–Meier curve for overall survival in patients with cStage III/IV disease. The analysis was performed in the overall survival cohort limited to patients treated between January 2017 and December 2023. RAMIE, robot-assisted minimally invasive esophagectomy; VATS, video-assisted thoracoscopic esophagectomy. (**b**) Kaplan–Meier curve for recurrence-free survival in patients with cStage III/IV disease. The analysis was performed in the overall survival cohort limited to patients treated between January 2017 and December 2023. RAMIE, robot-assisted minimally invasive esophagectomy; VATS, video-assisted thoracoscopic esophagectomy.

To further address the imbalance in clinical stage and to evaluate whether the numerically favorable OS after RAMIE was confined to a specific stage group, additional cStage-stratified exploratory survival analyses were performed in the overall survival cohort. In patients with cStage 0/I/II disease, OS and RFS did not differ significantly between VATS and RAMIE (3-year OS, 81.8% vs. 85.4%, log-rank *p* = 0.839; 3-year RFS, 69.2% vs. 75.5%, log-rank *p* = 0.584; Figure 3a,b). In patients with cStage III/IV disease, OS was significantly higher after RAMIE than after VATS (3-year OS, 48.5% vs. 65.6%, log-rank *p* = 0.019; Figure 4a), whereas RFS was comparable between groups (3-year RFS, 34.1% vs. 39.7%, log-rank *p* = 0.530; Figure 4b).

## 4. Discussion

In this updated single-institution analysis limited to one-stage gastric conduit reconstruction, RAMIE demonstrated short-term outcomes comparable to VATS with respect to overall postoperative morbidity, severe complications, anastomotic leakage, pneumonia, surgical site infection, operative time, blood loss, and postoperative hospital stay. The principal finding was that RLNP was less frequent after RAMIE, and this difference persisted after PSM that included preoperative therapy and the extent of lymph node dissection. In the exploratory PSM survival cohort, OS and RFS did not differ significantly between VATS and RAMIE, suggesting that RAMIE did not compromise long-term oncologic outcomes, although this analysis was not powered to prove oncologic equivalence.

These findings should be interpreted alongside existing randomized and institutional evidence. In Japan, thoracoscopic esophagectomy is an established standard comparator supported by multicenter randomized data [3]. The RAMIE trial showed comparable perioperative safety between RAMIE and conventional MIE, and the REVATE trial demonstrated improved dissection success along the left RLN and less postoperative RLNP after robotic surgery [7,8]. The present study differs from these randomized trials because it reflects real-world practice at a Japanese high-volume center where VATS was already established. Compared with our previous three-arm institutional report, the present analysis excluded open surgery, expanded the RAMIE cohort, incorporated preoperative therapy into PSM, and added exploratory survival analyses [10]. Thus, our study is intended to complement, rather than supersede, randomized evidence.

The reduction in RLNP after RAMIE is biologically plausible because robotic assistance can support stable visualization and precise instrument motion during standardized upper mediastinal lymphadenectomy. Our standardized RAMIE procedure emphasizes preservation of the RLNs in their original position, early division of esophageal branches, precise counter-traction, and limited use of energy devices close to the nerve [16]. This interpretation is consistent with prior reports evaluating RLNP in robot-assisted/conventional MIE and nerve-preserving minimally invasive lymphadenectomy [18,19]. After matching, the proportions of 3FLND and preoperative therapy were well balanced, yet RLNP CD grade >= I and CD grade >= II remained less frequent after RAMIE. Nevertheless, 3FLND includes cervical lymphadenectomy, and traction or injury to the vagus nerve or RLN during the cervical phase can contribute to postoperative vocal cord dysfunction independently of the robotic thoracic procedure; therefore, the 2FLND/3FLND subgroup findings should be interpreted as supportive but not definitive evidence that the thoracic robotic platform itself prevents RLNP.

The cStage-stratified exploratory analyses provide an additional perspective on oncologic outcomes. In cStage 0/I/II disease, OS and RFS were similar between VATS and RAMIE, whereas in cStage III/IV disease, OS was significantly higher after RAMIE while RFS remained comparable. A recent report of RAMIE for highly locally advanced cT3 borderline/cT4b esophageal cancer similarly showed no significant disease-free survival difference but better 3-year OS after RAMIE than after conventional MIE [20]. These findings raise the hypothesis that RAMIE may be particularly useful in advanced disease, possibly through precise upper mediastinal lymphadenectomy around the RLNs. However, because RFS was not significantly different and the stage-stratified analyses were not separately propensity score-matched, this finding should be interpreted cautiously as hypothesis-generating.

The discrepancy between OS and RFS in cStage III/IV disease may also reflect postoperative functional preservation rather than improved recurrence control alone. Previous studies have shown that RLNP after esophagectomy is associated with postoperative pneumonia and dysphagia and that dysphagia can be associated with pneumonia after discharge and long-term nutritional deterioration [21,22]. Dysphagia and impaired airway protection may also contribute to sarcopenic dysphagia, and sarcopenia has been associated with postoperative pneumonia and worse long-term survival after esophagectomy [23,24]. Therefore, the lower incidence of RLNP after RAMIE may help preserve airway protection, swallowing function, nutritional status, skeletal muscle mass, and postoperative performance status, potentially improving tolerance to post-recurrence treatment and reducing non-cancer-related mortality. These mechanisms remain clinically plausible but were not evaluated directly in the present retrospective dataset, and future prospective studies should examine them using longitudinal functional, nutritional, sarcopenia, recurrence-site, and post-recurrence treatment data.

This study has several limitations. It was retrospective and conducted at a single institution; the treatment approach was not randomized, and residual confounding cannot be excluded despite PSM using major clinicopathological, treatment-related, and surgical factors. The choice between VATS and RAMIE may have been influenced by patient selection, surgeon experience, team learning, and robotic system availability. The study period included the introduction and standardization of RAMIE; nevertheless, RAMIE showed comparable overall short-term safety and lower RLNP even with these early implementation cases included. Learning-curve and era-related effects should therefore be considered when interpreting the results. In this context, learning-curve and implementation studies in minimally invasive esophagectomy underscore the importance of structured standardization and accumulated experience when interpreting comparative outcomes [25,26]. Detailed surgeon-specific case volume, formal learning-curve analyses, platform-specific comparisons between da Vinci Xi and hinotori systems, harvested lymph-node counts including RLN stations, thoracic-phase operative time and blood loss, lymph-node recurrence patterns, RLNP recovery, swallowing rehabilitation, aspiration, tracheostomy, reintubation, cost-effectiveness, performance status, nutritional trajectory, skeletal muscle mass, cause-specific death, immune checkpoint inhibitor use, and detailed post-recurrence treatment were not available. Because aspiration and swallowing dysfunction can be influenced by RLNP as well as sarcopenia, frailty, pulmonary reserve, and other postoperative events, future prospective studies should evaluate these factors together to clarify their contribution to functional recovery and survival. The stage-stratified survival analyses were exploratory, not separately propensity score-matched within each stage subgroup, and limited by event number; therefore, the significantly higher OS observed after RAMIE in cStage III/IV disease requires validation. Future multicenter prospective studies should incorporate standardized surgeon credentialing, platform-specific reporting, detailed lymph-node harvest and recurrence data, functional RLNP outcomes, economic evaluation, and at least 5-year follow-up [9,25,26,27].

## 5. Conclusions

RAMIE was associated with comparable overall short-term safety to VATS in patients undergoing one-stage gastric conduit reconstruction for esophageal or esophagogastric junction cancer. RAMIE showed a lower incidence of RLNP in the overall cohort and after PSM that accounted for the clinicopathological factors, preoperative therapy, reconstruction route, abdominal approach, and lymphadenectomy extent. Exploratory long-term analyses suggested that RAMIE did not compromise oncologic outcomes. In cStage III/IV disease, OS was significantly higher after RAMIE whereas RFS remained comparable, supporting further investigation of the potential oncologic and functional value of precise robotic mediastinal lymphadenectomy in advanced disease. These findings should be validated in prospective multicenter studies with longer follow-up.

## Figures and Tables

**Table 1:** Baseline characteristics before and after propensity score matching.

Characteristic	Main Cohort			PSM Cohort		
	VATS (*n* = 186)	RAMIE (*n* = 183)	*p* Value	VATS (*n* = 128)	RAMIE (*n* = 128)	SMD
Age, years	69 (63.2–75)	70 (63–76)	0.569	69 (63–75.2)	70 (63–76)	0.001
Male sex	164/186 (88.2%)	157/183 (85.8%)	0.497	113 (88.3%)	109 (85.2%)	0.092
BMI, kg/m^2^	21.2 (19.1–23.8)	21.8 (19.9–24.4)	0.059	21.7 (20–24.1)	21.4 (19.3–23.8)	0.066
Histology			0.040			0.073
SCC	150 (80.6%)	128 (69.9%)		94 (73.4%)	98 (76.6%)	
Adenocarcinoma	34 (18.3%)	49 (26.8%)		32 (25.0%)	29 (22.7%)	
Other	2 (1.1%)	6 (3.3%)		2 (1.6%)	1 (0.8%)	
Tumor location, Ut/Mt/Lt/Jz	17/75/63/31	22/70/44/47	0.059	15/54/33/26	15/52/34/27	0.032
cStage, 0/I/II/III/IV	2/53/51/70/10	4/51/32/75/21	0.058	2/36/28/54/8	3/37/26/53/9	0.056
cStage III–IV	80/186 (43.0%)	96/183 (52.5%)	0.069	62 (48.4%)	62 (48.4%)	0.000
Preoperative therapy			0.949			0.056
Upfront surgery	109 (58.6%)	107 (58.5%)		75 (58.6%)	78 (60.9%)	
Preoperative chemotherapy	72 (38.7%)	72 (39.3%)		50 (39.1%)	48 (37.5%)	
Preoperative chemoradiotherapy	5 (2.7%)	4 (2.2%)		3 (2.3%)	2 (1.6%)	
Extent of LND			<0.001			0.067
3FLND	86 (46.2%)	46 (25.1%)		44 (34.4%)	40 (31.2%)	
2FLND	100 (53.8%)	137 (74.9%)		84 (65.6%)	88 (68.8%)	
Reconstruction route			0.548			0.000
Posterior mediastinal	165 (88.7%)	168 (91.8%)		116 (90.6%)	116 (90.6%)	
Retrosternal	18 (9.7%)	12 (6.6%)		10 (7.8%)	10 (7.8%)	
Antethoracic	3 (1.6%)	3 (1.6%)		2 (1.6%)	2 (1.6%)	
Abdominal approach			0.038			0.054
Open	63 (33.9%)	44 (24.0%)		30 (23.4%)	33 (25.8%)	
Laparoscopy	123 (66.1%)	139 (76.0%)		98 (76.6%)	95 (74.2%)	

Data are presented as median (interquartile range), number (%), number/total number (%), or counts. *p* values are shown for the main cohort, and SMDs are shown for the propensity score-matched cohort. Clinical stage was classified according to TNM 8th. SMD, standardized mean difference; an absolute SMD < 0.1 was considered to indicate adequate covariate balance. BMI, body mass index; Jz, esophagogastric junction cancer; LND, lymph node dissection; SCC, squamous cell carcinoma; TNM, tumor-node-metastasis; Ut, upper thoracic; Mt, middle thoracic; Lt, lower thoracic. Preoperative chemotherapy includes patients who received systemic chemotherapy before surgery for initially unresectable or borderline resectable disease; preoperative chemoradiotherapy includes patients who underwent surgery after definitive chemoradiotherapy.

**Table 2:** Intraoperative and postoperative outcomes.

Outcome	VATS (*n* = 186)	RAMIE (*n* = 183)	*p* Value
Operation time, min	543 (506–583)	553 (508.5–598.5)	0.134
Anesthesia time, min	640 (589–687)	641 (595.5–693)	0.530
Blood loss, mL	140 (80–290)	129 (70–230.5)	0.337
Postoperative hospital stay, days	23 (20–32)	23 (19–29.8)	0.259
Overall complications CD ≥ II	61/186 (32.8%)	60/183 (32.8%)	0.999
Severe complications CD ≥ III	16/186 (8.6%)	22/183 (12.0%)	0.280
RLNP CD ≥ I	18/186 (9.7%)	7/183 (3.8%)	0.025
RLNP CD ≥ II	7/186 (3.8%)	1/183 (0.5%)	0.034
Anastomotic leakage CD ≥ II	17/186 (9.1%)	24/183 (13.1%)	0.224
Pneumonia CD ≥ II	24/186 (12.9%)	17/183 (9.3%)	0.269
Surgical site infection CD ≥ II	7/186 (3.8%)	5/183 (2.7%)	0.577

Data are presented as median (interquartile range) or number/total number (%). CD, Clavien–Dindo classification; RLNP, recurrent laryngeal nerve paralysis.

**Table 3:** Propensity score-matched short-term outcomes.

Outcome	VATS (*n* = 128)	RAMIE (*n* = 128)	*p* Value
Operation time, min	544 (507.5–582)	558 (507.5–599.2)	0.171
Anesthesia time, min	638 (588.5–684.5)	646.5 (591.2–693)	0.500
Blood loss, mL	125 (75–295.5)	132 (70–227.8)	0.773
Postoperative hospital stay, days	23 (19–29)	22 (19–30)	0.710
Overall complications CD ≥ II	42/128 (32.8%)	42/128 (32.8%)	1.000
Severe complications CD ≥ III	11/128 (8.6%)	14/128 (10.9%)	0.528
RLNP CD ≥ I	14/128 (10.9%)	4/128 (3.1%)	0.015
RLNP CD ≥ II	6/128 (4.7%)	0/128 (0.0%)	0.013
Anastomotic leakage CD ≥ II	9/128 (7.0%)	16/128 (12.5%)	0.141
Pneumonia CD ≥ II	15/128 (11.7%)	12/128 (9.4%)	0.542
Surgical site infection CD ≥ II	5/128 (3.9%)	5/128 (3.9%)	1.000

Data are presented as median (interquartile range) or number/total number (%). The PSM model included age, sex, BMI, histology, tumor location, clinical stage (0–II vs. III–IV according to TNM 8th), preoperative therapy, extent of LND, abdominal approach, and reconstruction route. CD, Clavien–Dindo classification; LND, lymph node dissection; PSM, propensity score matching; RLNP, recurrent laryngeal nerve paralysis.

**Table 4:** Propensity score-matched sensitivity analysis for recurrent laryngeal nerve paralysis according to lymphadenectomy extent.

Analysis	VATS	RAMIE	*p* Value
2FLND subgroup: RLNP CD ≥ I	4/84 (4.8%)	3/88 (3.4%)	0.654
3FLND subgroup: RLNP CD ≥ I	10/44 (22.7%)	1/40 (2.5%)	0.006
3FLND subgroup: RLNP CD ≥ II	6/44 (13.6%)	0/40 (0.0%)	0.015

CD, Clavien–Dindo classification; LND, lymph node dissection; RLNP, recurrent laryngeal nerve paralysis.

## Data Availability

The datasets generated and/or analyzed during the current study are not publicly available because they contain patient-level clinical information but are available from the corresponding author on reasonable request, subject to institutional approval.

## Abbreviations

2FLND, two-field lymph node dissection; 3FLND, three-field lymph node dissection; BMI, body mass index; CD, Clavien–Dindo classification; CI, confidence interval; EGJ, esophagogastric junction; LND, lymph node dissection; OS, overall survival; PSM, propensity score matching; RAMIE, robot-assisted minimally invasive esophagectomy; RFS, recurrence-free survival; RLN, recurrent laryngeal nerve; RLNP, recurrent laryngeal nerve paralysis; SCC, squamous cell carcinoma; SMD, standardized mean difference; VATS, video-assisted thoracoscopic esophagectomy.
